# Three-Dimensional Printed Porous PLA Scaffolds with Dual Functionality: Cell Proliferation Enhancement and Antibacterial Properties

**DOI:** 10.3390/polym17141928

**Published:** 2025-07-13

**Authors:** Renad N. AlQurashi, Noora M. Bataweel, Mehal Atallah AlQriqri, Sarah H. Alqahtani, Ahmad A. Basalah, Laila A. Damiati

**Affiliations:** 1 Department of Biological Sciences, Collage of Science, University of Jeddah, Jeddah 21589, Saudi Arabia; 2 King Fahd Medical Research Centre, King Abdulazizi University, Jeddah 21589, Saudi Arabia; 3 Regenerative Medicine Unit, King Fahd Medical Research Centre, King Abdulazizi University, Jeddah 21589, Saudi Arabia; 4 Advanced Diagnostics and Therapeutics Institute, Health Sector, King Abdulaziz City for Science and Technology (KACST), Riyadh 11442, Saudi Arabia; 5 Mechanical Engineering Department, Collage of Engineering and Architecture, Umm Al-Qura University, Makkah 21955, Saudi Arabia

**Keywords:** 3D printing, PLA scaffolds, porosity, mechanical properties, cell proliferation, bacterial adhesion

## Abstract

Scaffold architecture plays a significant role in regulating cellular and microbial interactions in tissue engineering applications. This study evaluates the performance of 3D-printed poly (lactic acid) (PLA) scaffolds with varying porosity levels (20%, 40%, 60%, 80%, and 100%) in mechanical strength, supporting human skin fibroblast (HSF) viability and reducing bacterial colonization of Gram-positive bacteria (*Staphylococcus epidermidis, Staphylococcus aureus*), and Gram-negative bacteria (*Pseudomonas aeruginosa, Escherichia coli*). The maximum tensile strength (28 MPa) was achieved in the 100% dense scaffold. Increasing porosity drastically decreased tensile strength, where 80% PLA scaffold possessed 16 MPa strength. At greater levels of porosity (60% and 40%), tensile strengths greatly decrease (8 MPa and 4 MPa), while ductility increases, especially at high porosity levels. HSF viability, assessed using the AlamarBlue assay, showed a time-dependent increase in cell proliferation, with the highest viability observed on scaffolds with 60% and 80% porosity. SEM imaging confirmed strong cell adhesion on the 80% porous scaffold, indicating that intermediate-to-high porosity enhances cell attachment and metabolic activity. In contrast, bacterial adhesion showed species-specific responses to scaffold porosity. *S. epidermidis* and *E. coli* exhibited a progressive increase in adherence with porosity, peaking at 100%. *P. aeruginosa* showed maximum adhesion at 80%, suggesting a porosity “sweet spot” that favors its colonization. *S. aureus* adhered most strongly to scaffolds with intermediate porosities (40–60%) and significantly less at 100% porosity. The current study provides insights into scaffold design considerations, emphasizing the need for optimized scaffold architecture that balances regenerative potential with infection control in tissue engineering applications.

## 1. Introduction

Polylactic Acid (PLA) has gained attention in the field of tissue engineering due to its characteristics such as biodegradability, biocompatibility, mechanical tunability, making PLA suitable for biomedical scaffold fabrication [1]. As a thermoplastic aliphatic polyester derived from renewable sources, PLA offers a favorable degradation profile and low cytotoxicity, making it a good choice for various applications such as wound healing, dermal repair, and bone regeneration [2,3,4]. In the context of skin tissue engineering, PLA-based scaffolds serve as structural and biological templates that mimic the extracellular matrix (ECM), supporting cellular adhesion, proliferation, and matrix deposition [5]. A recent study has demonstrated the versatility and effectiveness of PLA-based scaffolds in skin tissue engineering and wound healing. Novel electrospun PLA fiber scaffolds loaded with encapsulated polyepicatechin (PEC) physical gels were synthesized and evaluated for their interaction with human dermal fibroblasts (HDFs). The results showed that the addition of PEC increased the scaffolds’ antioxidant activity, mechanical strength, and initial cell survival, indicating their potential in regenerative medicine applications. Interestingly, human dermal fibroblasts (HDFs) cultured on the PLA/PEC scaffolds showed better metabolic activity and survival rates compared to those cultivated on pure PLA. This suggests that the bioactive properties of PEC positively influence cell attachment and growth during the early stages of culture [6].

Another investigation compared the biological behavior of HDFs on 3D-printed PLA, polycaprolactone (PCL), and polyethylene terephthalate (PET) scaffolds. The findings revealed that 3D-printed PLA scaffolds provided a more favorable environment for cell viability, adhesion, and uniform distribution than PCL and PET. HDFs on PLA scaffolds maintained their tissue-specific spindle morphology and showed stronger type IV collagen expression, which is crucial for skin regeneration. These results highlight the biocompatibility and regenerative potential of PLA in skin tissue engineering [7]. Furthermore, Kayadurmus et al. explored the use of 3D-printed PLA scaffolds loaded with whey protein concentrate (WPC) for wound dressing applications. The addition of WPC improved the swelling capacity, degradation rate, and cell viability of the scaffolds, with the highest cell viability observed in PLA scaffolds containing 50% WPC after seven days. The study also demonstrated that the release of WPC from the scaffold could be controlled, offering potential for tailored drug delivery in wound care. These results suggest that PLA/WPC composite scaffolds could serve as customizable and bioactive wound dressings for chronic skin injuries [8].

Three-dimensional printing involves creating three-dimensional objects by adding subsequent layers of material from a digital file [9]. The 3D-printed polymeric scaffolds have become a necessity in tissue engineering, considering their crucial role in fabrication to specific tissue types, such as bone, cartilage, skin, and vasculature, and can be designed to incorporate bioactive factors to enhance the regeneration of tissues [10,11,12]. As discussed by Donate et al. synthetic polymers, e.g., PLA, have stable and standard properties that can be modified in a relatively easy way during their industrial production [13]. Furthermore, studying scaffold design is significant due to its effect on the function of scaffolds. Several aspects must be considered, including external geometry, porosity and pore size, and mechanical properties [14]. Computational techniques integrated with 3D printing have transformed tissue engineering scaffold design. This permits the development of intricate microstructures with enhanced precision and control, facilitating cell growth, proliferation, and differentiation. Zhang et al. illustrated that the computational methods help forecast scaffold characteristics and refine designs, while 3D printing ensures reliable accuracy and reproducibility [15]. A key parameter influencing the biological performance of these scaffolds is porosity. Optimal porosity ensures sufficient surface area, interconnected pores for nutrient diffusion, oxygen diffusion, waste removal, and space for tissue ingrowth, all of which are essential for effective tissue engineering applications [16]. Furthermore, higher porosity typically enhances cell infiltration and vascularization but may compromise mechanical strength if not carefully optimized. On the other hand, low porosity may hinder nutrient diffusion and cell penetration, impeding tissue regeneration [17]. Therefore, fine-tuning scaffold porosity is essential for achieving a functional balance between biological performance and structural integrity. Moreover, besides influencing cell behavior, scaffold porosity also plays a critical role in microbial interactions. The increasing incidence of infections associated with tissue engineering applications, particularly in the context of wound repair, highlights the urgent need for innovative strategies to enhance the efficacy of scaffolding materials. PLA, while biocompatible, does not inherently possess antibacterial properties. As such, surface architecture, including porosity, roughness, and wettability, can be strategically manipulated to reduce bacterial adhesion and colonization [18,19].

Researchers have explored various innovative strategies to improve the antibacterial and biocompatible performance of PLA surfaces fabricated via additive manufacturing. In one approach, PLA plates produced by 3D printing were coated with a sealant containing silver nitrate crystals. This coating significantly reduced bacterial growth, achieving at least a 64-fold reduction in colony-forming units of both *E. coli* and *S. aureus* within 30 min compared to uncoated controls. The effectiveness was attributed to the microporous structure of the sealant, which facilitated the release of silver ions, while the varnish alone showed no inherent antibacterial effect [20]. In a complementary strategy, the surface of PLA matrices was modified using ultra-short femtosecond laser pulses to create hierarchical micro- and nano-structures. This laser patterning enabled precise control over surface roughness and wettability, thereby shifting the material from a hydrophobic to a super hydrophilic state. This structuring also promoted mesenchymal stem cell adhesion and orientation. The same roughened surfaces also increased the adhesion of *S. aureus*, highlighting the need for balance between cell compatibility and bacterial resistance in scaffold design [21]. Together, these studies demonstrate that both chemical (silver-based sealants) and physical (laser structuring) modifications of 3D-printed PLA can significantly impact their antibacterial and biological properties, providing tailored solutions for biomedical applications [20,21].

Conventional tissue healing methods, such as gauze, cotton, and bandages, lack inherent biological activity and may not adequately address the complexities of various types of injuries. They also cannot regulate the controlled release of bioactive components, a characteristic feature of advanced dressings like 3D-printed scaffolds [22]. Furthermore, traditional wound healing materials have often fallen short of sufficient healing, resulting in scar formation [23]. Thus, there is an urgent need for improved tissue engineering approaches, such as 3D-printed scaffolding, to overcome these limitations. Three-dimensional-printed scaffolds offer several advantages over traditional wound healing methods. They can be customized, flexible, and precise, enabling the creation of artificial tissue constructs with accurate geometric configurations that match tissue defects [4,24]. Consequently, using 3D-printed scaffolds represents an advanced and superior approach to tissue healing compared to conventional methods. On the other hand, the ability of 3D-printing technology to fabricate intricate, patient-specific structures with exceptional accuracy establishes its significance as a critical resource across a multitude of biomedical domains. These scaffolds can be engineered not only to support skin regeneration but also to address emerging clinical challenges such as antibiotic resistance and chronic wound infection [25]. Moreover, 3D-printed PLA scaffolds offer a significant cost advantage over conventional tissue healing methods. The material cost for PLA filament is relatively low and the production process allows for efficient and customizable fabrication without substantial additional expense [26]. In contrast, conventional wound dressings and advanced therapies often require frequent changes and increased labor, resulting in cumulative costs ranging from hundreds to thousands of dollars per patient [27].

While PLA-based scaffolds have been widely studied for their biocompatibility and regenerative characteristics, few investigations have evaluated how different porosity impact the dual response of microbial colonization and host viability within the same scaffold platform. Given these considerations, this study aims to evaluate the effect of 3D-printed polymeric scaffolds with different porosity on bacterial growth inhibition, as well as investigating the impact of these models on human skin fibroblast cell proliferation and viability. By establishing a relationship between scaffold architecture and biological performance, this research seeks to guide the rational design of PLA with different porosity for advanced skin tissue engineering applications.

## 2. Materials and Methods

### 2.1. Surface Fabrication

**Figure 1:**
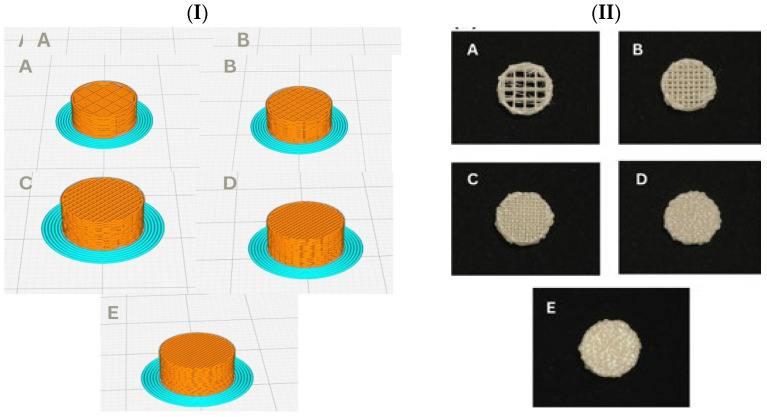
(**I**) CAD model of samples with different porosity percentages. (**II**): Images of the actual scaffolds after printing (A-20%, B-40%, C-60%, D-80%, E-100% porosity, respectively).

The material used in the study to fabricate samples was commercial PLA Push Plastic Filament (Push Plastic, Springdale, AR, USA). The 3D models of a rounded disk with dimensions of Ø 10.8 × 5 mm were generated with the assistance of the CAD package of the Craftware Pro v1.1.4.95 (Craftunique Kft., Budapest, Hungary). As shown in Figure 1(I), the designed models were sliced using the Ultimaker Cura v4.13.1 and exported into 2D layers in a g-code format to 3D printing machine. The fabrication of PLA samples was conducted by an Ultimaker3 3D Printer (Utrecht, Netherlands). In the printing process, samples were printed with varied levels of density: 20%, 40%, 60%, 80% and 100% with *n* = 8 for each group as shown in Figure 1(II). Material manufacturer recommendations were followed, including 205 °C printing temperature, 60 °C plate temperature, 50 mm/s as a printing speed and 0.2 mm as a layer thickness with a nozzle diameter of Ø 0.4mm.

### 2.2. Mechanical Test (Tensile Test)

**Figure 2:**
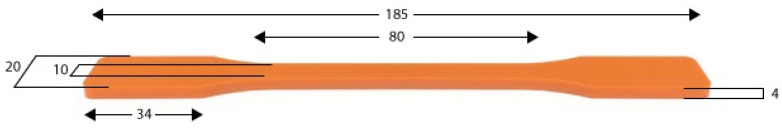
Dimensions of tension specimens of PLA specimen, ASTM D638.

The tensile properties of PLD material were determined according to the American Society for Testing and Materials D638 (ASTM). The test specimens were fabricated by using a 3D printer in the standard dimension in accordance with ASTM D638. The dimensions of the test specimens are illustrated in Figure 2.

**Figure 3:**
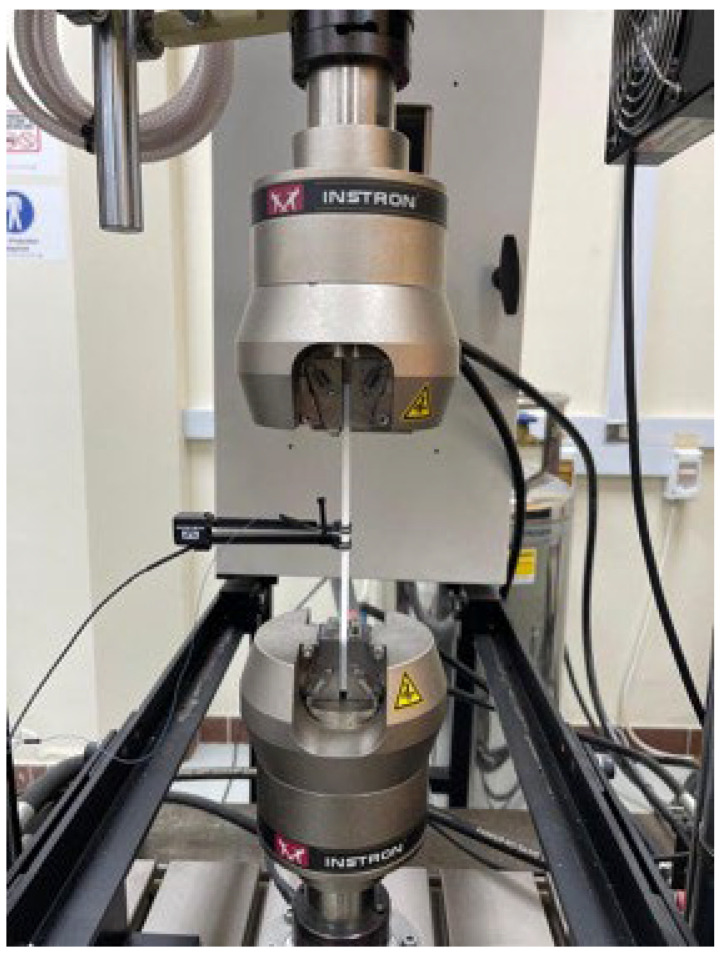
Experimental setup for tension tests.

The tensile tests were performed on the Instron servohydraulic universal testing machine model 8872 (10 kN) in an ambient laboratory environment. The capacity of the selected testing machine (10 kN) is very suitable for testing PLA materials, which have a maximum tensile load less than 1.2 kN. The longitudinal strain was recorded during the tensile test using the Extensometer model Instron 2620-601(Norwood, Massachusetts, USA) with an 80 mm gauge length as shown in Figure 3. The tension tests were implemented at a constant crosshead speed of 1.0 mm/min. At least four test specimens were tested for each material type. Most of the test specimens failed in the gage sections, which satisfied the conditions for selection width, thickness, and length of the ASTM standard.

### 2.3. Biological Tests

#### 2.3.1. Cell Culture

Human Skin Fibroblast (HSFs) (ATCC, Manassas, VI, USA) were cultured in DMEM supplemented with 10% (*v*/*v*) FBS, 1% (*v*/*v*) MEM NEAA (non-essential amino acid), 1% (*v*/*v*) 200 mM L-glutamine and 2% (*v*/*v*) antibiotics (6.74 U/mL penicillin-streptomycin) (Sigma-Aldrich, Dorset, UK) in T25 flasks. Flasks were incubated at 37 °C in 5% CO_2_ environment. For seeding, cells were rinsed twice in PBS saline, and then incubated with 1.5 mL trypsin (37 °C in 5% CO_2_) for 2–3 min. Next, 3 mL culture media was added to stop the action of the trypsin; the resulting cell suspension was transferred into 15 mL falcon tubes and centrifuged for 4 min at 200× *g* to obtain sediment cells. The trypsin/media supernatant was decanted, and the cells were resuspended in media. The cells were either seeded into new flasks or used for the experimental setup. In addition, 50,000 cells/scaffold were used in the rest of the experiments.

#### 2.3.2. AlamarBlue Assay

AlamarBlue is a commercial product (Bio-Rad, Watford, UK) used to test viability or growth of cells using a redox indicator, i.e., the reduction of resazurin to the resorufin product. The reduction results in a color change from blue to pink analyzed by a fluorometer at 560 nm excitation and 600 nm emission wavelength. The AlamarBlue solution was mixed 1:10 in DMEM; 1 mL was added to each scaffold and incubated for 6h (37 °C, 5% CO_2_). The supernatant was transferred to a 96-well plate in triplicate (3 × 200 μL) and measured using a plate reader (SpectraMax^®^ i3 Multi-Mode Microplate Detection Platform, San Jose, CA, USA).

#### 2.3.3. Scanning Electron Microscope (SEM)

After 24 h in culture, the samples were fixed in 1.5% glutaraldehyde buffer for 1h at room temperature (RT) and then rinsed in 0.1 M sodium cacodylate. They were post-fixed in 1% osmium tetroxide for 1 h at RT and then washed 3× with distilled H_2_O for 10 min. For dehydration, a 30–100% ethanol series was applied. A hexamethyldisiloxane step was conducted prior to sputter coating (20 nm palladium). To evaluate the cell morphology on PLA scaffolds, SEM was applied at a 5 kV accelerated voltage in the Secondary Electron Detector (SED) mode (SEM-6010 PLUS/LA, JOEL Inc., Peabody, MA, USA).

#### 2.3.4. Bacterial Culture

*Staphylococcus epidermis* (ATCC 29213), *Staphylococcus aureus* (ATCC 12600), and *Pseudomonads aeruginosa* (ATCC 1744), and *Escherichia coli* (ATCC 25922) were cultured in a Muller Hinton broth media and incubated overnight at 37 °C; for each design, scaffolds were placed in 24-well plates and seeded with 2 mL of bacterial suspension and incubated for 24 h at 37 °C. After 24 h, the scaffolds were transferred into 15 mL and then washed gently with phosphate-buffer saline (1× PBS), before being discarded. Afterwards, 1 mL of PBS was added to each tube containing the scaffolds and vortexed for 1 min. The optical density (OD) of adhered bacteria was measured at 600 nm using a UV–vis spectrophotometer (Thermo Scientific Genesys 10S UV–Vis) while applying PBS as the blank. In addition, the OD of each scaffold’s supernatant was measured.

#### 2.3.5. Bacterial Metabolomic Activity Measurement (ATP Release)

The BacTitre-GloTM assay (Promega, Southampton, UK) was used to determine the cell viability of bacteria cultured on different PLA scaffolds. Porous PLA scaffolds were placed on 24-well plates and submerged with the bacterial suspension (10^4^ CFU/mL). The plate was incubated for 24 h at 37 °C under static conditions. After 24h, the supernatant was moved to 96-well plates to measure the ATP release on the floating cells. The scaffolds were rained to remove non-adherent cells by gentle washing with 1× PBS. The scaffolds were transferred into bijou tubes with 1 mL 1× PBS and sonicated for 10 min to detach the adherent cells. The detached cells in suspension were transferred into 96-well plates. An equal amount of BacTitre-Glo regent was added, and the plates were read within 5 min of adding reagent using luminesce plate reader (SpectraMax^®^ i3 Multi-Mode Microplate Detection Platform, San Jose, CA, USA).

### 2.4. Statistical Analysis

Experiments were performed as three independent replicates and the results were presented as means ± standard errors (SDs). All statistical analyses were performed using GraphPad Prism V.10. Data were analyzed using Ordinary One-Way ANOVA, and *p*-values < 0.05 were considered significant.

## 3. Results and Discussion

### 3.1. Effect of Varying Levels of Porosity on the Mechanical Strength of the 3D-Printed Samples

**Figure 4:**
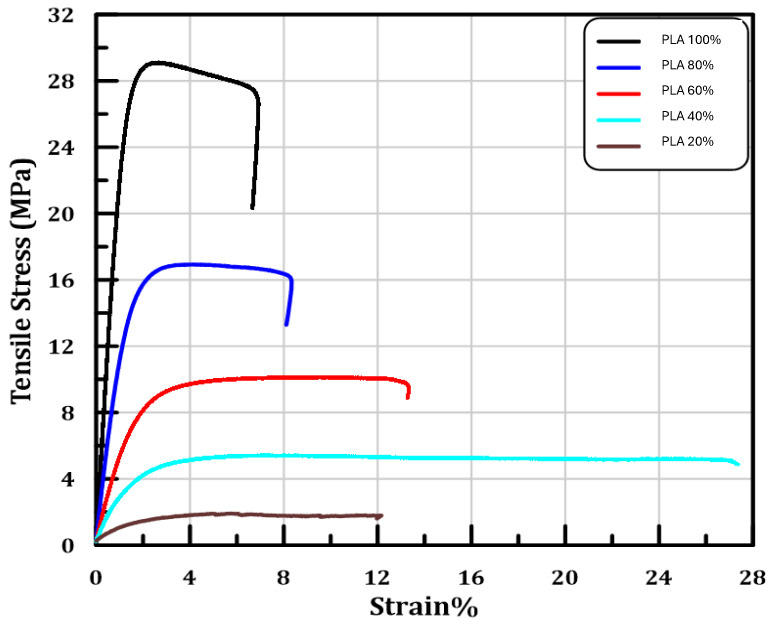
Stress–Strain relationship for PLA tension tests.

Tensile testing according to (ASTM D638) was employed to evaluate the mechanical properties of the PLA scaffolds of different porosities (20%, 40%, 60%, 80%, 100%). The stress–strain diagram for the five samples is presented in (Figure 4). The ultimate tensile strength (Ø) was calculated by dividing the ultimate tensile load Pult by the cross-section area (A) of gauge section as follows:

(1) $$\sigma =\frac{P_{ult}}{A}$$

The true modulus (*E_T_*) was calculated from the slope of the initial linear portion of the stress–strain curves as follows:

(2) $$E_{T}=\frac{\sigma }{\varepsilon_{extensometer}}$$

where Ø extensometer is the recorded strain of the extensometer.

Stress–strain curves described show significant variations in mechanical behavior of samples due to varying porosities. For the 100% fully dense PLA samples, tensile strength was the highest; however, brittle behavior was observed leading to failure at a strain of approximately 6%. This sample does not wear but is under the condition where mechanical strength is the highest at the expense of low ductility, demonstrating high stiffness values typical for pure PLA. For higher porosity (80% PLA) samples, a noticeable decrease in tensile strength was observed, about 16 MPa. The sample exhibited slightly higher ductility in spite of its lower strength than that of the fully dense PLA; the fracture strain increased to about 8%. The results indicate that increasing the porosity also increases the flexibility slightly; however, this reduces tensile strength. Interestingly, a further increase in porosity (60% PLA) resulted in a reduction in the tensile strength to ~8 MPa. The strain at fracture was approximately 12%, demonstrating slightly higher ductility than the 80% PLA sample; however, significantly lower strength was reported, indicating an interplay between mechanical integrity and porosity, which is favorable for some biomedical applications where mechanical performance is required but better permeabilities are needed. Samples with the highest levels of porosity, 40% and 20% PLA, exhibited low tensile strength, i.e., ~4 MPa and less than 2 MPa, respectively. These samples demonstrated significant plastic deformation with the 40% PLA with sustained strain capacity (~25%) prior to failure and the 20% PLA with levels of ductility (12%). The substantial drop in tensile strength at high porosities highlights the mechanical trade-off of creating such large voids, a design that is well-suited for applications where flexibility and cell penetration are more critical than mechanical strength.

These findings match with previous reports. A study by Chen et al. showed that the mechanical support (Young’s modulus) of PLA scaffolds (64.8% porosity) was proportional to porosity and deformation under loads, showing predictable strength reduction at higher porosities [28]. Similarly, Liao et al. illustrated a clear linear correlation between porosity and elastic modulus. As porosity increases, the elastic modulus decreases. In thick-layer samples with ~32% porosity, the elastic modulus dropped significantly, although post-printing heat treatment could enhance crystallinity and partially recover mechanical properties [29]. Overall, the mechanical tests reveal a well-defined correlation between the porosity and the mechanical properties, where increasing the porosity lowers the tensile strength and raises the ductility. Thus, the choice of scaffold must take into account the specific tissue engineering application, weighing together mechanical strength, suppleness, and bioactivity.

### 3.2. Cell Culture and Visualization

**Figure 5:**
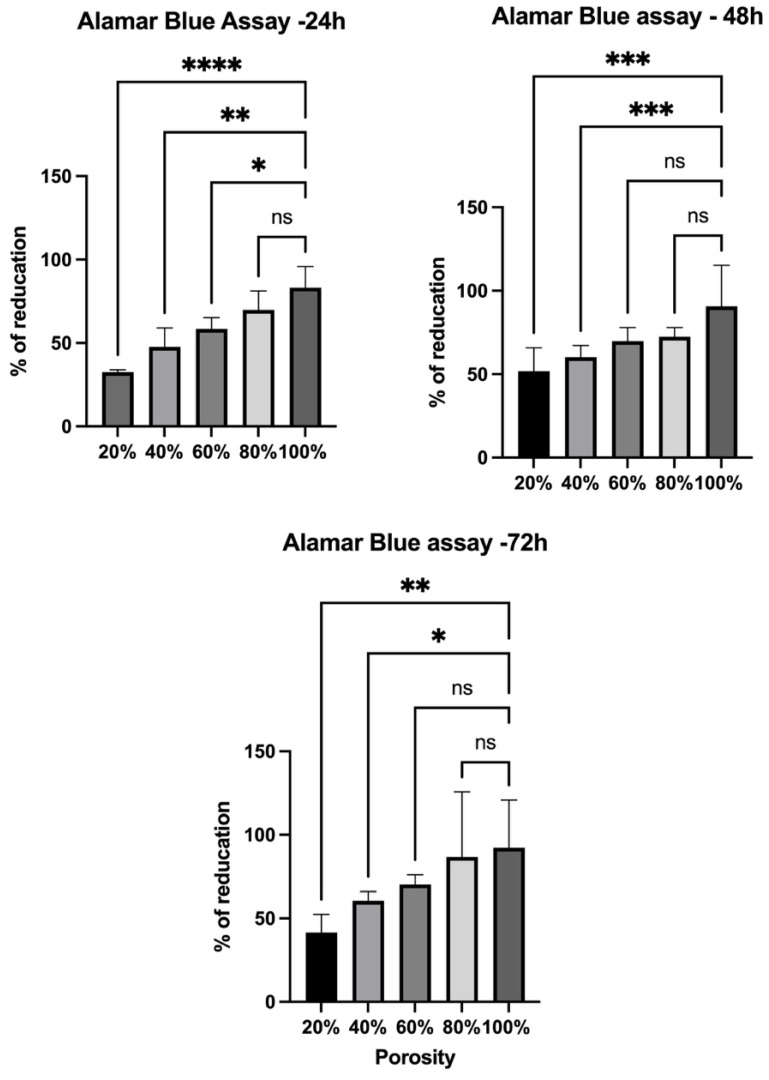
Viability of HSF cultured on scaffolds with different porosities assessed by the AlamarBlue assay after 24–72 h. Results demonstrate time-dependent increases in cell viability across all groups, with scaffolds possessing 60% and 80% porosity and supporting the highest proliferation rates, compared to the control porosity of 100%. Data presented as mean ± SD. Statistical analysis was performed using One-Way ANOVA test (* *p* < 0.5, ** *p* < 0.01, *** *p* < 0.001, **** *p* < 0.0001, ns = non-significant), where *n* = 3.

**Figure 6:**
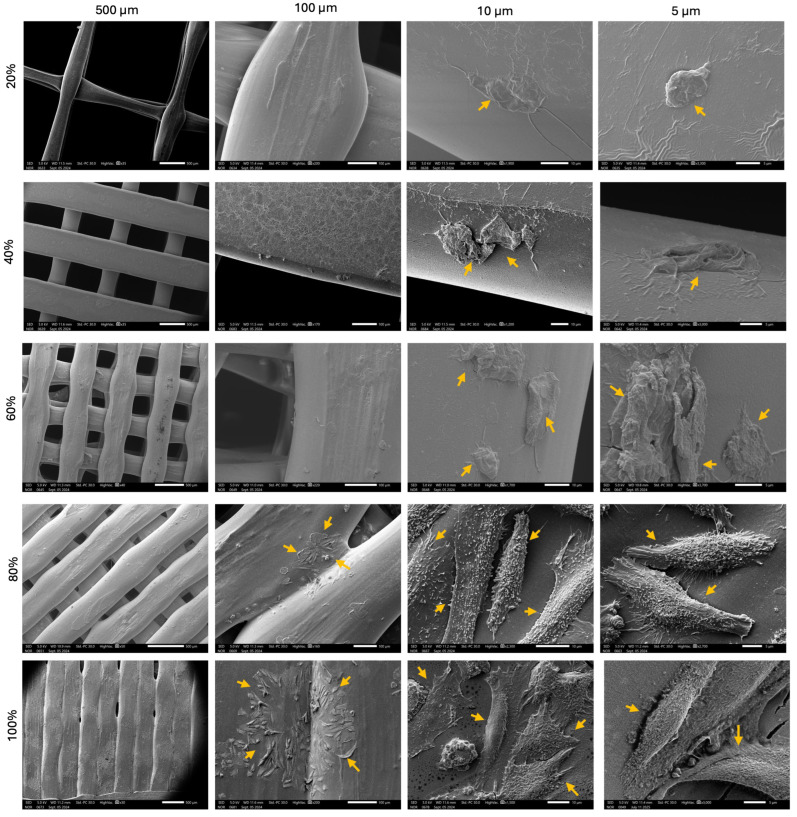
SEM images of HSFs cultured on PLA scaffolds with varying porosities. The image shows HSF adherence and cell spreading across scaffolds (yellow arrows) with 20%, 40%, 60%, 80%, and 100% porosity. The scaffolds with higher porosity (80% and 100%) demonstrate improved cell adhesion and enhanced spreading, with cells appearing more elongated and distributed uniformly on the surface. In contrast, scaffolds with lower porosity show reduced cell attachment and limited spreading, indicating that higher porosity promotes better cellular interactions with PLA scaffolds.

Cell viability of human skin fibroblasts was assessed using the AlamarBlue assay at 24, 48, and 72 h (Figure 5) and demonstrated a time-dependent increase across all scaffold porosities. The highest proliferation was observed on scaffolds with 60% and 80% porosity, indicating that intermediate-to-high porosity levels provide more favorable conditions for cell attachment and growth. In contrast, lower porosity scaffolds (20%) showed reduced viability, likely due to limited surface area and nutrient diffusion. These findings matched with the SEM images (Figure 6). Our findings also matched with previous reports, including a study by Mandal and Kundu that illustrated that the pore sizes of 200 to 250 mm and porosity of approximately 86% enabled better HSF cell proliferation on 3D silk fibroin scaffolds [30]. Generally, the large surface area-to-volume ratio of nanofibrous meshes enhances cell attachment and proliferation, which is consistent with our HSF viability results showing increased fibroblast proliferation on higher-porosity scaffolds [31,32]. These findings indicate that scaffold architectures offering greater accessible surface areas provide a more favorable environment for cell–matrix interactions, thereby promoting efficient wound closure and tissue regeneration.

### 3.3. Bacterial Culture

**Figure 7:**
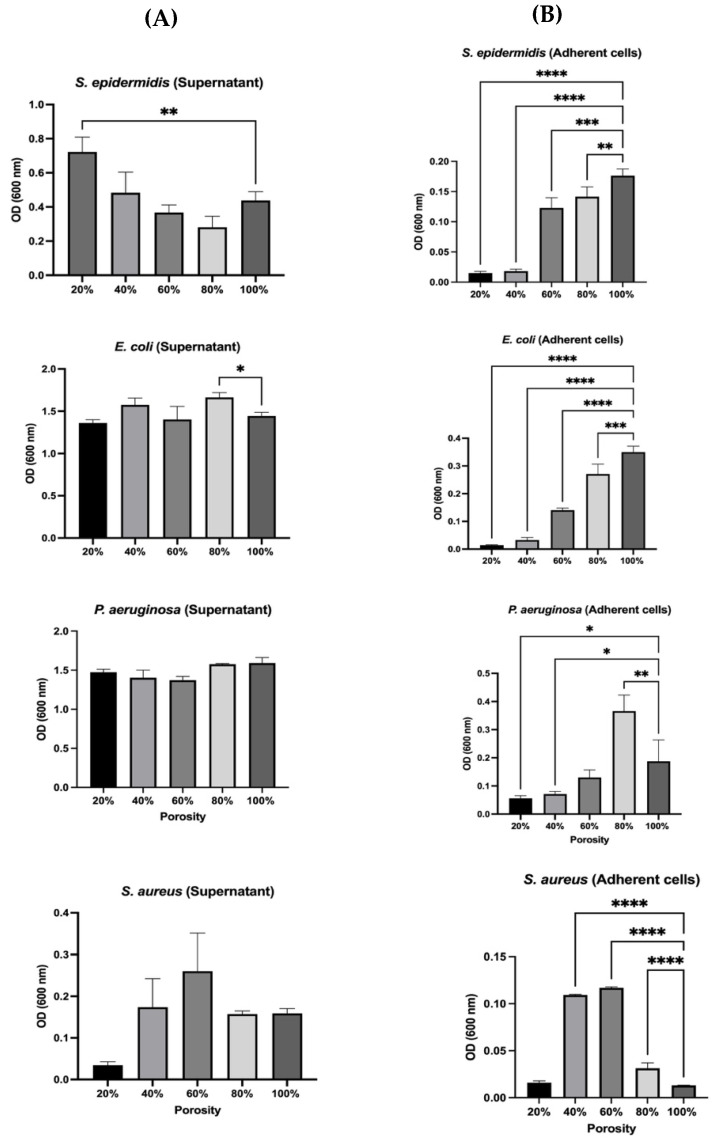
Optical density (O.D) measurements of bacterial supernatants after incubation on scaffolds with varying porosity levels (20%, 40%, 60%, 80%, and 100%) for four bacterial strains: *S. epidermidis, E. coli, P. aeruginosa*, and *S. aureus*. (**A**) These measurements reflect planktonic bacterial populations in the surrounding media. Variations in O.D indicate species-specific responses to scaffold porosity, with trends suggesting differences in growth, detachment, or metabolic state. (**B**) Results show the porosity-dependent adhesion patterns, with *S. epidermidis* and *E. coli* exhibiting increased adhesion at higher porosity (peaking at 100%), while *P. aeruginosa* shows maximal adhesion at 80%. S. aureus displays greatest adhesion at intermediate porosities (40% and 60%). Data are presented as mean ± SD. Statistical analysis was performed using One-Way ANOVA test (* *p* < 0.5, ** *p* < 0.01, *** *p* < 0.001, **** *p* < 0.0001), where *n* = 3.

In order to determine the adherence of bacteria on PLA scaffolds, Figure 7 shows the optical density (OD) of adhered bacteria for each design that was measured. As can be seen in Figure 7A, the O.D of the planktonic (free-floating) for the *S. epidermidis* scaffold with 20% porosity contained the highest number of bacterial cells, whereas the scaffold with 80% porosity exhibited the lowest bacterial population. Scaffolds with 40% and 100% porosity showed similar bacterial counts, with a moderate decrease observed at 60% porosity. This indicates that lower porosity retains more bacteria in the medium, while higher porosity may allow better adhesion or entrapment. For both *E. coli* and *P. aeruginosa* supernatant O.D, bacterial growth followed a consistent trend across all porosity levels. Meanwhile, the scaffold with 80% porosity showed a slight increase in the *E. coli* population, which was unusual compared to the overall trend. The unexpected increase for *E. coli* at 80% porosity may suggest altered surface interaction or biofilm formation. For *S. aureus* supernatant O.D, the highest bacterial population was demonstrated at 60% porosity. The scaffolds with 40%, 80%, and 100% porosity supported similar levels of bacterial growth, while the 20% porosity scaffold resulted in the lowest growth of *S. aureus*. Peak O.D was reported at 60% porosity, which may suggest favorable conditions for planktonic growth, while the lowest was reported at 20%, which may indicate stronger attachment or poor survival in the medium. On the other hand, as shown in Figure 7B, the number of adhered bacterial cells generally increased with scaffold porosity, starting from the lowest at 20% and peaking at 100% for *S. epidermidis*; this trend was particularly evident for *E. coli*, which mirrored the overall pattern. This suggests that higher porosity enhances surface area and nutrient exchange, thereby promoting colonization. However, *P*. *aeruginosa* demonstrated a significant increase in cell numbers when cultured on scaffolds with 80% porosity, a trend that was not observed at lower porosity levels of 20%, 40%, and 60%. In the case of *S. aureus*, the highest bacterial population was observed at 60% porosity and 40%. The 80% and 20% porosity scaffolds supported similar bacterial numbers, while the 100% porosity scaffold exhibited the lowest *S. aureus* growth. Overall, for *S. epidermidis* and *E. coli*, adhesion increased progressively with porosity, peaking at 100%, suggesting that higher porosity enhances surface area and nutrient exchange, thereby promoting colonization. *P. aeruginosa* exhibited a distinct adhesion peak at 80% porosity, indicating a possible porosity “sweet spot” that optimally supports its attachment, which did not persist at 100%. In contrast, *S. aureus* showed maximum adhesion at intermediate porosities (40% and 60%), while adhesion significantly decreased at 100% porosity, possibly due to reduced surface retention or altered topography at extreme porosity levels. These species-specific trends highlight the critical role of scaffold architecture in modulating microbial behavior.

**Figure 8:**
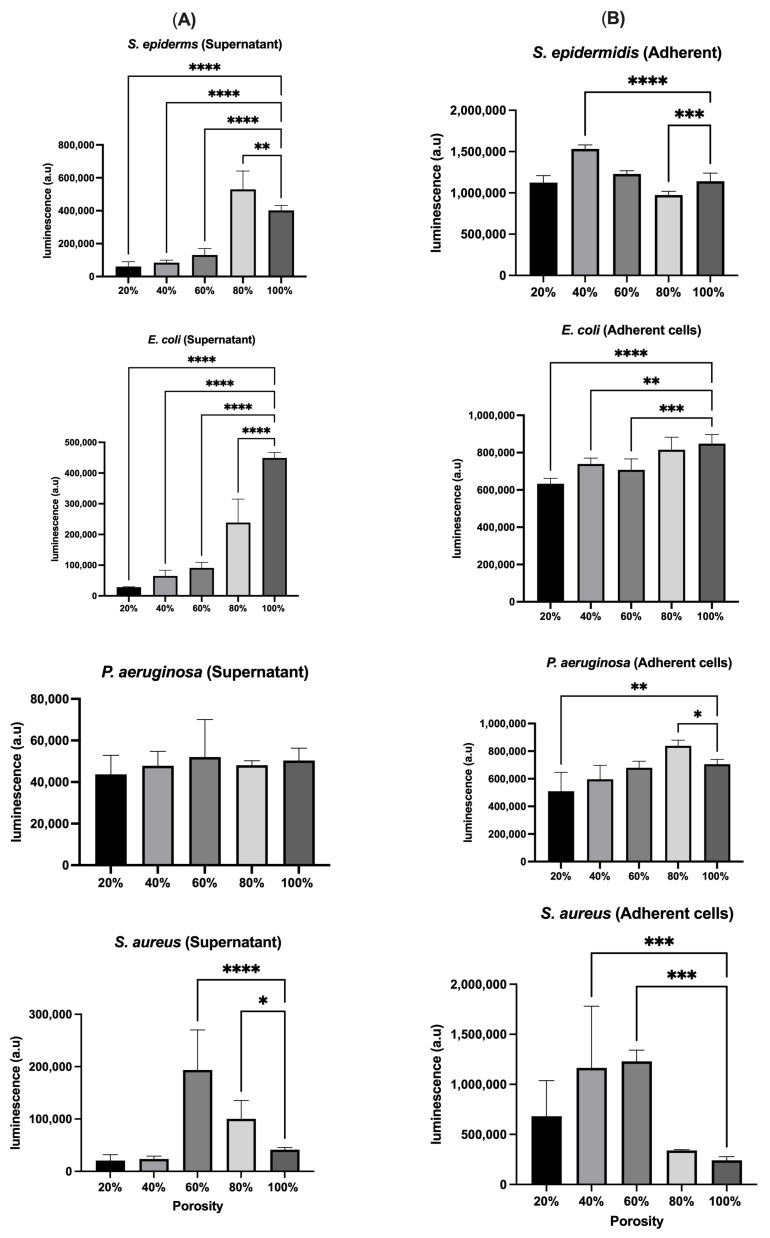
Luminescence measurements of bacterial supernatants following incubation on scaffolds with different porosity levels. (**A**) Luminescence intensity reflects the metabolic activity and viability of planktonic (free-floating) bacterial cells. Differences in luminescence across porosities indicate species-specific viability responses, with *S. epidermidis* and *E. coli* showing enhanced activity at higher porosities, while *S. aureus* exhibited variable results not fully consistent with optical density trends. (**B**) Luminescence analysis of bacteria adhered to the scaffold surfaces. *S. epidermidis* and *E. coli* showed increased viability with increasing porosity, although luminescence values differed from corresponding OD-based adhesion patterns. *P. aeruginosa* demonstrated a notable increase in adherent cell viability at 80% porosity, while *S. aureus* showed similar luminescence trends to OD results. Data are presented as mean ± SD. Statistical analysis was performed using One-Way ANOVA test (* *p* < 0.5, ** *p* < 0.01, *** *p* < 0.001, **** *p* < 0.0001), where *n* = 3.

Figure 8A illustrates the metabolic viability of planktonic bacteria using luminescence assays across scaffolds with varying porosities. *S. epidermidis* exhibited the lowest viability at 20% porosity and the highest at 80%, a trend that contrasts with OD measurements, suggesting OD may include non-viable cells. *E. coli* showed peak luminescence at 100% porosity, confirming increased metabolic activity and viability with higher porosity. For *P. aeruginosa*, luminescence trends mirrored OD results, with an exception at 60% porosity, where elevated luminescence indicated a potential metabolic advantage. In the case of *S. aureus*, luminescence data did not fully align with OD results, particularly at 40% and 100% porosity, where OD indicated high biomass, but luminescence revealed lower viability. On the other hand, Figure 8B presents the metabolic activity of scaffold-adherent bacterial cells measured by luminescence. For *S. epidermidis*, 40% porosity yielded higher luminescence than expected from OD data, while 20% porosity showed low luminescence, indicating a high proportion of non-viable adherent cells. *E. coli* luminescence trends generally matched OD results, although viability was higher than OD indicated at lower porosities. *P. aeruginosa* demonstrated consistent increases in adherent viability at 80% porosity across both luminescence and OD, reinforcing this as an optimal porosity for colonization. For *S. aureus*, luminescence results closely aligned with OD values, confirming the scaffold’s role in modulating both adhesion and cell viability.

Generally, the comparative analysis between OD and metabolomic activity measurements revealed notable discrepancies in bacterial quantification across different scaffold porosities, particularly for *S. epidermidis*, *E. coli*, and *S. aureus*. While OD reflects the total biomass, including live, dead, and metabolically inactive cells, luminescence specifically indicates metabolically active, viable bacterial populations. For instance, *S. epidermidis* exhibited high OD at low porosity (20%), yet low luminescence, suggesting the presence of non-viable or dormant cells. In contrast, *E. coli* showed higher luminescence than OD at higher porosities (80–100%), indicating increased metabolic activity despite moderate total biomass. These findings highlight the importance of integrating both analytical methods to accurately assess bacterial colonization and viability, as OD alone may overestimate live bacterial populations in bioengineered environments (Table 1).

These findings align with previous studies that demonstrate that surface architecture plays an important role in modulating cellular and microbial responses on PLA scaffolds. A study by Daskalova et al. demonstrates that the ultra-short laser modification of PLA surfaces has the ability to generate different hierarchical microstructures by adjusting the laser influence and scanning velocity, which results in significant changes in surface roughness, porosity and wettability. The increased porosity in PLA scaffolds may not only enhance nutrient diffusion and cell proliferation but also affect bacterial colonization patterns. It was found that *S. aureus* showed higher adhesion onto PLA samples treated with the laser patterning type (G2) in comparison to the control (G3) scaffolds [21]. Another study by Rocha et al. illustrated that the silver nitrate crystal sealant on PLA scaffolds has a significant impact on the inhibition of bacterial growth of *E. coli* and *S. aureus* compared to unsealed scaffolds by at least 64 times in 30 min. This may be due to its microporous structure, which likely served as a conduit for silver ions from the encapsulated silver nitrate to exert their bactericidal effect on bacterial colonies [20]. Furthermore, a study by Lanno et al. showed that porous chloramphenicol-loaded polycaprolactone (PCL) microfiber scaffolds were more elastic compared to nonporous scaffolds and had the highest antibiofilm activity against *E. coli* and *P. aeruginosa*. These findings indicate that the pores on single fibers within an electrospun scaffold, in addition to the nano- and microscale diameter of the fibers, change the living cell−fiber interactions that affect the antibiofilm efficacy [33]. Taking into account the fact that scaffold porosity plays an important role in drug release, a study by Chen et al. demonstrated that coaxial polycaprolactone (PCL)/PLA core–shell porous nanofibers loaded with Roxithromycin, an antibacterial agent, exhibited effective antimicrobial activity. The inhibition zone diameter of the coaxial nanofibers with two different pore sizes was 1.70 ± 0.10 cm and 1.73 ± 0.23 cm, exhibiting a good antibacterial effect against *S. aureus* [34]. The next table (Table 2) summarizes recent work on PLA-based scaffolds regarding cellular and bacterial behavior.

Our findings also reinforce the limitations of OD-based bacterial quantification. The relationship between OD and actual cell number is not always linear, particularly at higher OD values (>1), and can be influenced by cell size, aggregation, and spectrophotometer characteristics [35,36]. Bacterial byproducts may increase light absorption, further complicating OD interpretation. Variations in bacterial size, morphology, and growth patterns—including clumping or chain formation—disrupt the correlation between OD and cell concentration, as some bacteria are smaller than 600 nm and scatter poorly, while others aggregate unpredictably [37]. Therefore, multi-wavelength analysis is recommended for more accurate bacterial quantification, as it minimizes the impact of byproducts and morphological variation on the optical signal [38].

## 4. Conclusions

In this study, we demonstrated that scaffold porosity plays an important role in influencing both bacterial colonization and HSF viability in PLA-based 3D-printed constructs. Quantification of adherent bacteria by OD and luminescence assays revealed species-specific trends, with *S. epidermidis* and *E. coli* showing a progressive increase in adhesion as porosity increased, peaking at 100%. *P. aeruginosa* exhibited maximum adhesion at 80% porosity, suggesting a porosity “sweet spot” that favors its colonization, while *S. aureus* adhered most strongly to scaffolds with intermediate porosities (40–60%) and showed significantly reduced adhesion at 100%. These findings indicate that higher scaffold porosity enhances surface area and nutrient exchange, promoting microbial colonization—but the response varies by bacterial species. On the other hand, fibroblast viability increased over time and was the highest on scaffolds with intermediate to high porosity (60–80%), underscoring the role of porosity in facilitating nutrient diffusion, waste removal, and cellular infiltration. These findings were consistent with the principle that larger surface area-to-volume ratios, as seen in porous or nanofibrous structures, enhance cellular interactions and support wound healing. Furthermore, high porosity was found to negatively impact the mechanical strength of the PLA scaffolds, with high porous structure (>80%) exhibiting reductions in the tensile test and structure integrity compared to lower porosity counterparts. It is important to note that the porosity alone does not fully capture the complexity of scaffolds’ architecture. Specifically, the surface area available for cell and microbial interactions is influenced not only by porosity percentage but also by pore size, shape, and spatial, distribution. Higher porosity does not always equate to higher surface area, especially when scaffold geometry varies. More analysis incorporating surface-area-to-volume ratio would offer deeper insights into the biological response observed. Overall, the results confirm that modifying scaffold porosity can strategically balance host cell compatibility and microbial resistance, making PLA scaffolds with optimized architecture promising candidates for advanced skin tissue engineering and infection-responsive wound dressings. However, this study has few limitations. For instance, the mechanical evaluation was limited to tensile testing under static conditions, and future work could include more comprehensive mechanical and degradation analysis. In addition, while in vitro assays revealed porosity-dependent trends in cell viability and bacterial adhesion, in vivo validation is required to confirm these observations in complex biological systems. Future studies may also investigate the incorporation of antibacterial agents to further improve resistance to infection without affecting biocompatibility.

## Figures and Tables

**Table 1:** Summarizing bacterial responses to different PLA scaffold porosities across OD and luminescence assays.

Bacteria	OD (Planktonic)	OD (Adherent)	Luminescence (Planktonic)	Luminescence (Adherent)	Conclusion
*S. epidermidis*	High at 20%, low at 80%	Increases with porosity, peaks at 100%	High at 80%, low at 20%	High at 40%, low at 20%	OD may include non-viable cells; luminescence confirms viability at 80%
*E. coli*	Consistent trend, slight increase at 80%	Increases with porosity, peaks at 100%	Highest at 100%	Generally matches OD, higher at low porosity	High metabolic activity at high porosity
*P. aeruginosa*	Consistent across porosities	Sharp increase at 80%, drops at 100%	Follows OD, peak at 60%	Consistent trend, peak at 80%	80% porosity optimal for attachment and viability
*S. aureus*	High at 60%, low at 20%	Highest at 40–60%, lowest at 100%	Mismatch with OD, lower at 40% and 100%	Aligns with OD	Discrepancy between OD and viability at extreme porosities

**Table 2:** Summary of previous work on PLA-based scaffolds.

Scaffolds/Odification	Bacterial Response	Cellular Response	Study
PLA + PEC (electrospun)	Not reported	Improved HDF viability and metabolic activity	[6]
Three-dimensional-printed PLA vs. PCL and PET	PLA superior to PCL and PET (general biocompatibility)	Higher HDF viability and collagen expression on PLA	[7]
PLA + WPC	Not specified	Enhanced viability, swelling, and degradability	[8]
PLA + silver coating	64x CFU reduction in *E. coli* and *S. aureus*	Not reported	[20]
Laser-structured PLA surface	Increased *S. aureus* adhesion	Promoted stem cell adhesion and orientation	[21]
Porous chloramphenicol-loaded PCL microfibers	Porous fibers showed highest antibiofilm activity and better elasticity than nonporous fibers	Not reported	[33]
Coaxial PCL/PLA porous nanofibers loaded with Roxithromycin	Porous nanofibers showed good antibacterial zones (1.70–1.73 cm) against *S. aureus*	Not reported	[34]
Three-dimensional-printed PLA scaffolds with varying porosities	Porosity influenced bacterial adhesion and viability; OD alone is insufficient for accuracy; luminescence revealed metabolic activity more accurately	HSF viability increased over time and was highest on scaffolds with intermediate to high porosity (60–80%)	Current study

## Data Availability

The original contributions presented in the study are included in the article; further inquiries can be directed to the corresponding author.

## Abbreviations

The following abbreviations are used in this manuscript:

PLA	Poly(lactic acid)
HSF	Human skin fibroblast
ASTM	American Society for Testing and Materials
ECM	Extracellular matrix
NEAA	Non-essential amino acid
RT	Room temperature
PBS	Phosphate-buffer saline
CFU	Colony-forming unit
OD	Optical density
PCL	Polycaprolactone
